# Deterioration of pulmonary function: An early complication in Fibrodysplasia Ossificans Progressiva

**DOI:** 10.1016/j.bonr.2021.100758

**Published:** 2021-02-25

**Authors:** Esmée Botman, Bernard J. Smilde, Max Hoebink, Sanne Treurniet, Pieter Raijmakers, Otto Kamp, Bernd P. Teunissen, Arend Bökenkamp, Patrick Jak, Adriaan A. Lammertsma, Joost G. van den Aardweg, Anco Boonstra, Elisabeth M.W. Eekhoff

**Affiliations:** a Amsterdam UMC, Vrije Universiteit Amsterdam, Department of Internal Medicine section Endocrinology, Amsterdam Movement Sciences, Amsterdam Bone Centre, de Boelelaan 1117, Amsterdam, the Netherlands; b Amsterdam UMC, Vrije Universiteit Amsterdam, Department of Radiology and Nuclear Medicine, de Boelelaan 1117, Amsterdam, the Netherlands; c Amsterdam UMC, Vrije Universiteit Amsterdam, Department of Cardiology, de Boelelaan 1117, Amsterdam, the Netherlands; d Amsterdam UMC, Emma Children's Hospital, Vrije Universiteit Amsterdam, Department of Pediatric Nephrology, de Boelelaan 1117, Amsterdam, the Netherlands; e Amsterdam UMC, Vrije Universiteit Amsterdam, Department of Pulmonology, de Boelelaan 1117, Amsterdam, the Netherlands

**Keywords:** Fibrodysplasia Ossificans Progressiva, Heterotopic ossification, Pulmonary function

## Abstract

Fibrodysplasia Ossificans Progressiva (FOP) is a genetic disease characterized by the formation of heterotopic ossification (HO) in connective tissues. HO first develops in the thoracic region, before more peripheral sites are affected. Due to HO along the thoracic cage, its movements are restricted and pulmonary function deteriorates. Because development of HO is progressive, it is likely that pulmonary function deteriorates over time, but longitudinal data on pulmonary function in FOP are missing.

Longitudinal pulmonary function tests (PFTs) from seven FOP patients were evaluated retrospectively to assess whether there were changes in pulmonary function during aging. Forced vital capacity (FVC), forced expiratory volume in one second (FEV1), total lung capacity (TLC), residual volume (RV) and diffusing lung capacity for carbon dioxide divided by alveolar volume (DLCO/VA) were included. In addition, HO volume along the thorax together with its progression as identified by whole body low dose CT scans were correlated to PFT data.

Per patient, aged 7–57 years at the time of the first PFT, three to nine PFTs were available over a period of 6–18 years. Restrictive pulmonary function, identified by TLC or suspected by FVC, was found in all, but one, patients. In three patients, TLC, FVC or both decreased further during the follow-up period. All, but one, patients had an increased RV. The DLCO/VA ratio was normal in all FOP patients. Interestingly, FEV1 increased after a surgical intervention to unlock the jaw. In four out of five patients total HO volume in the thoracic region progressed beyond early adulthood, but no further decline in FVC was observed.

In conclusion, restrictive pulmonary function was found in the majority of patients already at an early age. Our data suggest that the deterioration in pulmonary function is age dependent.

## 1 Introduction

Fibrodysplasia Ossificans Progressiva (FOP) is a rare, disabling genetic disease, which is characterized by the formation of heterotopic ossification (HO) in muscles, ligaments and tendons (Kaplan and Smith, 1997; Cohen et al., 1993). HO often is preceded by a flare-up, an inflammatory process with, as yet, unknown pathophysiology. In most cases the first flare-ups occur around the age of six, often involving neck and upper back (Pignolo et al., 2016). Thoracic HO immobilizes the thoracic cage, restricting normal expansion of the lungs (Kaplan et al., 2010; Kaplan and Glaser, 2005; Kussmaul et al., 1998; Connor et al., 1981). As a result, patients are dependent on diaphragmatic breathing as the diaphragm is spared in FOP (Connor et al., 1981). Mean life expectancy of patients with FOP is limited to 40–50 years of age, with cardiorespiratory complications as the major cause of death (Kaplan et al., 2010; Kaplan and Glaser, 2005). Some cross-sectional studies have shown restricted pulmonary function in FOP, which was attributed to limited chest mobility (Kussmaul et al., 1998; Connor et al., 1981; Buhain et al., 1974). It is not known, however, whether pulmonary function declines further while the disease is progressing. Longitudinal pulmonary function tests (PFTs) could give more insight on the impact of both HO volume and its progression on pulmonary function (Botman et al., 2019).

To the best of our knowledge, longitudinal data on pulmonary function in FOP patients have not been studied yet. It can be hypothesized that a decline in pulmonary function, if present, is related to (chronic) progression of HO around the thoracic cage. The aim of this study was therefore to assess the relationship between temporal changes in PFTs and volumetric HO changes along the thoracic area.

## 2 Methods

FOP patients treated at the FOP Expertise Center of Amsterdam UMC were included if successive PFTs were available between 1995 and 2019. In addition to PTFs obtained at the Amsterdam UMC, test results from referring centers were also included in the analysis.

Furthermore, whenever available, whole body low dose computed tomography (WBLDCT) scans were used to assess volume and progression of HO in the thoracic area and to evaluate structural changes within the lung parenchyma.

The Medical Ethics Review Committee of the Amsterdam UMC, Vrije Universiteit Amsterdam approved the study. All patients signed informed consent for the analysis and anonymous publication of their data.

### 2.1 Pulmonary function tests

nPFTs were obtained during yearly follow-up visits at Amsterdam UMC. Before 2014, spirometry, body plethysmography and single-breath transfer factor of the lung for carbon monoxide (DCLO) were measured using VMAX equipment from SensorMedics (Yorba Linda, CA, USA). From 2014, the Sentrysuite v.2.19 spirometry instrument (Carefusion, San Diego, California, USA) and the Vyntus body plethysmograph (Vyaire Medical, Mettawa, Illinois, USA) were used. Static lung volumes of patients who were unable to enter the plethysmograph because of wheelchair dependency, were assessed by nitrogen washout on the VMAX equipment and by helium wash-in on the SentrySuite instrument. The following pulmonary function parameters were measured: forced vital capacity (FVC), vital capacity (VC), forced expiratory volume in one second (FEV1), residual volume (RV), expiratory reserve volume (ERV) and total lung capacity (TLC). In addition, lung diffusion was assessed by measuring diffusing capacity for carbon dioxide (DLCO) where DLCO was divided by alveolar volume (DLCO/VA). Reference values used for static and dynamic lung parameters were taken from the Global Lung Function 2012 Equations (Quanjer et al., 2012). For DCLO and DCLO/VA the guidelines of the Global Lung Function Initiative 2017 were used (Stanojevic et al., 2017). FVC, TLC, FEV1, DLCO and DLCO/VA were expressed as percentage of predicted, based on gender, height and ethnicity according to those guidelines (Quanjer et al., 2012; Stanojevic et al., 2017). For all measures, a percentage of predicted below 80% was considered deviant and the Tiffeneau index (FEV1/FVC) was considered deviant when below 70% (Quanjer et al., 2012; Stanojevic et al., 2017).

### 2.2 Low dose CT-scans

Low-dose CT scans, acquired at 120 kV with a tube current ranging from 30 to 60 mAs, were assessed for structural abnormalities in lung parenchyma. CT scans were included when performed within either seven days prior to or one month after PFT. Images were analyzed by both a nuclear medicine specialist (PR) and a pulmonologist (AB) to assess parenchyma, pulmonary vasculature, pleurae, bronchi and heart size. Both, PR and AB, evaluated lung parenchyma blinded to the PFT results.

In addition, consecutive whole body low-dose CT scans (WBLDCT) were analyzed to identify HO lesions throughout the body and around the thoracic area. These HO lesions were identified and analyzed manually in order to calculate their volumes in successive images. Both readers (BT and EB) were blinded to the PFT of the patient. The association between HO volume of the total body, and HO within the thoracic area or the thoracic back and pulmonary function was statistically assessed. In addition, correlation between progression of HO in the thoracic area (chest) and decline in pulmonary function was evaluated.

Moreover, the presence and a kyphosis and scoliosis was assessed using the available CT-scans. Both the scoliosis and kyphosis angle was assessed using Cobbs Angle (Cobb and Cobb, 1948).

### 2.3 Statistical analysis

Statistical analyses were performed using SPSS Statistics for Windows, (IBM, version 24.0, Armonk). The Spearman's rho was used to assess correlations between HO volumes and change in PFT parameters.

## 3 Results

Longitudinal PFTs of seven FOP patients were included in the study. All patients, except one, had the classic mutation (R206H) (Table 1). The patient with the variant Q207E did not differ phenotypically from those with the classic mutation. All patients exhibited the classical clinical features of FOP with progressive HO formation. During flare-ups all patients underwent standard therapy with corticosteroids and non-steroidal anti-inflammatory drugs. Unfortunately, the frequency of flare-ups and drug treatments over the years had not been recorded. One patient (patient 004) is a smoker, with 5 pack years up to the end of the included period.

**Table 1:** Baseline characteristics and available data of the included FOP patients.

	Sex	Mutation	Age^a^	Follow-up (years)	BMI^b^	Number of PFTs	Successive WBLDCT
001	♂	R206H	12	8	27.2	6	Yes
002	♀	R206H	35	8	13.3	3	Yes
003	♀	R206H	21	6	20.2	4	Yes
004	♂	R206H	57	11	26.3	8	No
005	♂	R206H	21	6	24.2	4	No
006	♀	Q207E	14	8	27.2	3	Yes
007	♀	R206H	7	18	20.5	9	Yes

Abbreviations: FOP = fibrodysplasia ossificans progressiva; BMI = Body Mass Index; PFTs = pulmonary function tests; WBLDCT = Whole Body Low Dose Computed Tomography.

a At the time of the first pulmonary function test.

b Highest BMI measured during the follow-up period.

In total, 37 PFTs were included in the analysis. Of these PFTs, 12 were obtained in childhood (<18 years of age). Per patient, three to nine PFTs were available over a period of six to eighteen years. The age at which the first pulmonary function was obtained ranged from 7 to 57 years (Table 1). 61% of all the PFTs were obtained at Amsterdam UMC. In two patients, tests were performed in other centers.

In five patients at least two WBLDCT scans were available during the observation period to relate HO progression in the thoracic region with pulmonary function.

For all 37 PFTs, FEV1 and FVC were available, while TLC was only measured in 20 of the 37 PFTs (from 6 patients). Diffusion capacity was measured in 15 of the 37 PFTs from six patients.

### 3.1 Spirometry (FVC, VC, FEV1)

**Fig. 1:**
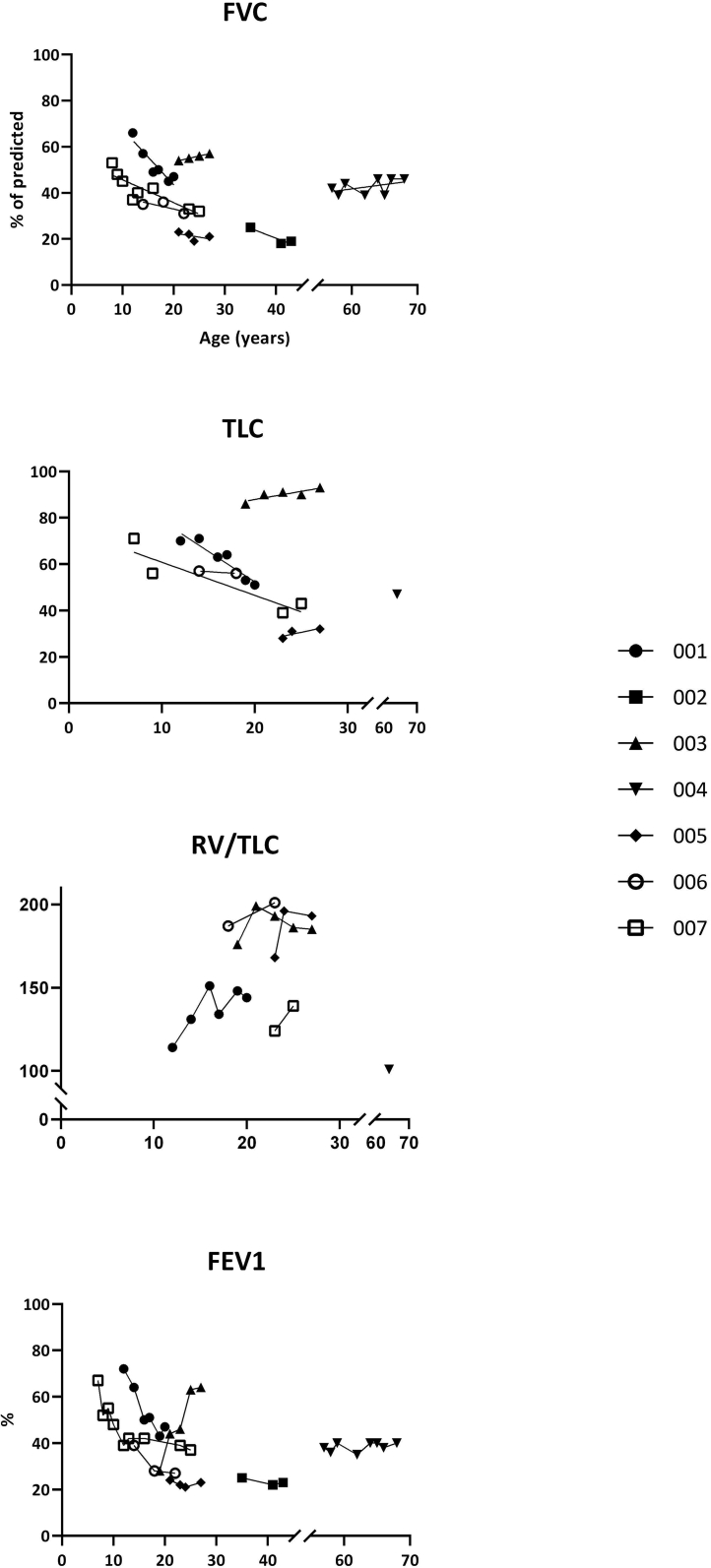
Longitudinal Pulmonary function test data of FOP patients A decrease in FVC was seen in patient 001, 006 and 007. The decline was already seen in childhood and early adolescence. TLC decreased in patient 001 and 007. The discrepancy between FVC and TLC in patient 003 was caused by an increased RV/TLC-ratio. This ratio was increased compared to predicted in all patients. Furthermore, FEV1 decreased over time in patient 001, 006 and 007. Patient 003 regained a few millimeters mouth opening after surgery, with an increase of FEV1 as result. TLC, FVC and the RV/TLC-ratio did not change due to the increased mouth opening. Abbreviations: FOP = fibrodysplasia ossificans progressiva; FVC = forced vital capacity; TLC = total lung capacity; RV = Residual Volume; FEV1 = forced expiratory volume in one second.

FVC was below 80% of predicted in all patients irrespective of age and was already <80% in the youngest patient tested at the age of seven. FVC deteriorated over time in three of the seven FOP patients with a decline already in childhood.(Fig. 1). In two of these patients, worsening continued into young adulthood, up to the age of twenty-three. For two other patients, also followed in their twenties, FVC did not increase or decline during observation. For the oldest two patients in this study, followed from the age of 57 to 68 years and 35 to 43 years, respectively, both absolute and percentage of predicted values for FVC remained stable. VC was congruent with FVC for all patients throughout the follow-up period.

FEV1/FVC, also known as the Tiffeneau index, was below 70% of predicted in two patients, aged 18 and 68 years old (Tiffeneau of 68% and 65%, respectively). For all other patients the Tiffeneau index was >70%, ruling out any obstructive component. Interestingly, one patient who underwent jaw surgery experienced an increase in the Tiffeneau index from 70% to 95% following surgery. The increased Tiffeneau index resulted from an increase in FEV1 (from 46% to 64%), possibly related to the slight improvement (3 mm) of mouth opening (supplemental data, Fig. 1). FVC and VC remained stable pre- and post-operatively. In addition, also RV did not change due to the increased mouth opening. The Tiffeneau index in five patients with and two patients without jaw ankyloses showed no obvious difference (65–93% vs 91–94%, respectively).

### 3.2 Static lung volumes (TLC, RV, ERV)

TLC follow-up data were available for six patients. For one patient, only one measurement was available. A low TLC, indicating restrictive lung function (below 80% of predicted) was observed in five of the six patients. One patient with a low FVC and VC, appeared to have a normal TLC (Fig. 1).

Deterioration of TLC during the observation period was observed in two patients during childhood and early adolescence. TLC in one patient decreased until the age of 19 and in the other patient between the age of 9 and 23. After the age of 23, no decline in TLC was found, but it should be noted that this is based on data of only two patients who were followed for up to 2 years after stabilization of TLC. For the two oldest patients, TLC follow-up data were not available.

The RV to TLC ratio was increased in five of the six patients (Fig. 1). The ratio ranged from 125 to 200% of predicted. The increase in mouth opening after surgery, did not result in normalization of RV. However, it did result in an increased volume of air that could be exhaled forcibly (ERV). Prior to surgery the ERV for this particular patient was 0.86 L, which increased to 1.16 L after surgery. For all other patients ERV ranged between 0.74 L and 0.1 L.

### 3.3 Diffusing capacity (DLCO and DLCO/VA)

DLCO was available for six patients and between 40 and 60% of predicted in five patients. The patient who underwent oro-maxillary surgery showed an no increase in DLCO after surgery. Also, no statistical difference in DLCO was found between patients with (four out of six patients) and without jaw ankyloses (two out of six patients) (Mann Whitney U: p = 0.64). When DLCO was corrected for alveolar volume (DLCO/VA), diffusion was >80% for all six patients.

### 3.4 Lung parenchyma

Low-dose CT scans to evaluate lung parenchyma were available for all seven patients. Five showed a highly deformed thoracic cage, while it was nearly normal in the other two patients. The presence and severity of kyphosis and scoliosis are presented in Table 2. These two patients were 20 and 24 years old. Both had normal lung parenchyma, no pleural thickening or cardiomegaly. Three of the five patients with a deformed thorax showed intrapulmonary abnormalities: one had a partial atelectasis of the lower left lobe of ≈330 mL, occupying 4% of the total lung volume. One had mild ground-glass opacity at the age of 22. There were no abnormalities in the parenchyma or pleurae. One patient, aged 65, had a minimal consolidation (3 mm), which is currently being followed by high resolution CT according to the Fleishner criteria (MacMahon et al., 2017).

**Table 2:** Demographics of the included FOP patients.

Patient	Age at diagnosis	Age et first flare-up	History of pneumonia?	Mobility (age of wheelchair dependence)	Kyphosis^a^ angle (°)	Scoliosis^a^ angle (°)
001	5	5	No	Ambulant	10	n/a
002	3	UNK	Yes	Wheelchair bound (19)	67	60
003	10	5	No	Ambulant	n/a	n/a
004	12	UNK	No	Wheelchair bound (48)	44	50
005	6	UNK	No	Wheelchair bound (21)	n/a	55
006	6	6	No	Wheelchair bound (20)	60	n/a
007	6	6	No	Wheelchair bound (18)	n/a	20

Age in years, angles in degrees.

Abbreviations: UNK = unknown; n/a = not applicable/absent.

a Kyphosis and scoliosis angles of the cervical and thoracic spine only were calculated, as this would impact pulmonary function.

### 3.5 Heterotopic ossification

In five patients successive LDWBCT-scans over a period of 6–26 months were available. Neither total body HO volume, HO volume within the thoracic area, nor HO volume along the thoracic back were significantly correlated with any of the PFT parameters (supplemental data). HO progression in the thoracic area was seen in four patients and ranged from 5 to 11 mL in 6 to 26 months. The most prominent progression in the thoracic area with an increase in HO of 11 mL in 6 months was accompanied by stable TLC, FVC and DLCO/VA (Fig. 1). Including all five patients for whom successive CT scans were available, no association was found between HO expansion and FVC changes over this short period of time (spearman's rho = −0.2; p = 0.7). In addition, no association was found between total HO volume, thoracic HO volume and the PFT parameters (supplemental data).

## 4 Discussion

Thirty-seven PFTs were analyzed in a longitudinal cohort of seven FOP patients to determine whether lung function over a period of 6 to 18 years was associated with HO volume and HO progression in the thoracic area. A restrictive pulmonary function was found in all but one patients. This restriction in pulmonary function deteriorated over time during childhood and early adolescence, but a further decline later in life was not observed. No significant obstructive pulmonary function was found, nor a relationship between the degree of pulmonary function impairment and thoracic HO volume.

FEV1 is the simplest parameter to obtain a rapid assessment of lung function. A reduced FEV1 value usually is related to the degree of obstructive pulmonary function (Pellegrino et al., 2005; Brazzale et al., 2016). In the current cohort, significantly reduced FEV1 values were found in all patients, but in the presence of a normal Tiffeneau index. The reduced FEV1 value can therefore be attributed to reduced lung volumes in FOP patients. An ankylosed jaw, often seen in FOP, affects FEV1 values. In one the present patients, FEV1 increased by 40% after surgical unlocking the ankylosed jaw. A similar effect on FEV1 was found in possibly the only published study on this topic, assessing the effect of maxillomandibular fixation in healthy subjects on pulmonary function, where FEV1 decreased with approximately 20% as a result of the maxillomandibular fixation (Kohno et al., 1993). In addition, a study simulating the effect of upper airway obstruction using mouth pieces with orifices of different diameters, found that FEV1 progressively worsened with decreasing diameters (Empey, 1972). Therefore, although FEV1 was decreased in most of the present FOP patients due to decreased lung capacity, jaw occlusion also may play a role in the severity of FEV1.

All patients had reduced FVC and VC, which suggests the presence of restrictive lung function. It should be noted, however, that to confirm restrictive pulmonary function initially, assessment of total lung volume (TLC) is essential, and therefore the use of an additional test is required (Pellegrino et al., 2005). In the present study, a normal TLC was only observed in one patient, despite a suggestive restriction based on FVC (57%). This may be explained by an increased RV, seen in all but one of the patients, and which represents the difference between TLC and VC. The underlying mechanism of an increased RV in FOP patients could be the inability to fully exhale, possibly due to completely ankyloses of the thoracic cage. An increased RV is also seen as an early pulmonary abnormality in neuromuscular diseases (Chiang et al., 2018). In later stages, muscle weakness leads to a restrictive pulmonary function. Whether this process also occurs in FOP patients, could not be confirmed with the current limited dataset. Remarkably, RV in the 65-year old patient was within the normal range. With aging, RV is thought to increase as a result of stiffening of the thoracic wall (Sharma and Goodwin, 2006). FOP patients will not be susceptible for this stiffening as they are already completely ankylosed, explaining the increased, but stable, RV throughout the observed period in the current cohort. As RV does not seem to fluctuate over time, FVC can be used to monitor pulmonary function after initial confirmation of restriction by TLC.

The restrictive pulmonary function, as seen in the present study, is in concordance with findings of two cross-sectional studies (Kussmaul et al., 1998; Connor et al., 1981). These two studies included 21 and 15 FOP patients, respectively. Unfortunately TLC values were not obtained. Although the age of the patients studied by Kussmaul et al. ranged from 5 to 55 years, values were only presented for the entire group, making it impossible to compare pulmonary function of younger patients with that of older patients (Kussmaul et al., 1998). On the other hand, On the other hand, in their study on adult FOP patients, Connor et al. concluded that age had no effect on the degree of restriction, as FVC did not differ between age groups (Connor et al., 1981). It remains uncertain whether such a plateau of deterioration does indeed occur and if so, at what age. We could only follow three patients from childhood to adolescence during a relatively short period. Longer follow-up and more structural PFTs are needed to confirm whether pulmonary function indeed stabilizes at a certain age. It is assumed that the restrictive pulmonary function is the result of both malformed costovertebral joints and chest wall deformities due to asymmetrical HO formation along the spine and thoracic cage (Kaplan et al., 2010). One could argue that the severity of the chest wall deformity (kyphosis or scoliosis) should have an impact on the restriction of the thoracic cage and therefore pulmonary function (Daghighi and Tropp, 2019; Farrell and Garrido, 2020). Two of the patients with relatively mild thoracic deformities did, however, show severe restricted pulmonary function. Therefore, thoracic deformity alone does not fully predict abnormality of lung function. In addition, the location of HO, especially whether HO is located near crucial joints or not, might also be important. Also, pulmonary function hardly appears to deteriorate later in life, it seems that the amount of HO formed in the thoracic area at younger age may already be sufficient to restrict pulmonary function and that it is not affected by further (later) progression of HO. In an attempt to further investigate this, effects of total amount and chronic growth of HO in the thoracic region and pulmonary function were assessed, but neither showed a relationship with the pulmonary function nor its decline. Moreover, previously it has been shown that chest wall expansion does not deteriorate further after the age of 15 (Kussmaul et al., 1998). However, lifetime PFT data from FOP patients are not available yet.

Life-long PFT data and WBLDCT images will be needed to evaluate the clinical relevance of mild abnormalities of the lung parenchyma, which were seen in two of the patients in the present study. Restrictive pulmonary function may lead to relaxation atelectasis, which may reduce TLC. In the present study no distinction was made between restriction caused by an immobile thorax or by atelectasis. Atelectasis observed in one of the patients covered only a small portion of total TLC and, therefore, thoracic stiffness is likely to be the most important cause of restrictive pulmonary function in FOP. In an attempt to prevent atelectasis, respiratory muscle training, using a hand-held unit with tubing and a connected rebreathing bag, could be considered in an attempt to maintain adequate ventilation of the alveoli (Budweiser et al., 2006). In addition, if oxygen therapy is considered, the patient should be monitored closely to prevent hypercapnia and hyperoxia related damage to airways or pulmonary parenchyma (Kaplan and Glaser, 2005), especially as, in general, patients with a severely affected, immobilized thoracic cage already have a degree of hypercapnia (Bergofsky, 1979).

The strength of this study is the long follow-up period of a group of FOP patients. The main limitation of the present study is the inability to relate pulmonary function tests to clinical data, due to incomplete documentation. Also, given that data were acquired within the context of standard patient care, PFTs had not been obtained regularly and not all parameters were obtained. In addition, measuring equipment was not standardized over time or between different centres. Throughout time various measuring instruments have been used, and, data of other centers are included of which the measuring instruments are unknown.

In conclusion, longitudinal PFTs confirmed restricted pulmonary function in FOP patients already at young age. TLC is necessary to confirm the restrictive component in FOP patients initially, as increased RV may affect FVC. In addition, it should be noted that FEV1 values are diminished due to small lung volumes, but might also be affected the jaw ankylosis. Neither total volume of HO nor progression of thoracic HO seemed to affect pulmonary function later in life. It will be clear, however, that longer follow-up periods are needed to confirm this finding.

The following are the supplementary data related to this article.

Supplementary Table 1 Volume of heterotopic ossification in relation to the pulmonary function. Supplementary Table 1

## Transparency document

Transparency document. Image 1

## CRediT authorship contribution statement

**Esmée Botman:** Conceptualization, Methodology, Investigation, Formal analysis, Resources, Visualization, Validation, Writing – original draft, Writing – review & editing. **Bernard J. Smilde:** Investigation, Formal analysis, Writing – review & editing. **Max Hoebink:** Conceptualization, Methodology, Investigation, Visualization, Writing – original draft. **Sanne Treurniet:** Methodology, Writing – review & editing. **Pieter Raijmakers:** Conceptualization, Methodology, Writing – review & editing. **Otto Kamp:** Conceptualization, Methodology, Writing – review & editing. **Bernd P. Teunissen:** Conceptualization, Methodology, Writing – review & editing. **Arend Bökenkamp:** Conceptualization, Methodology, Writing – review & editing. **Patrick Jak:** Conceptualization, Methodology, Validation, Writing – review & editing. **Adriaan A. Lammertsma:** Methodology, Investigation, Supervision, Writing – review & editing. **Joost G. van den Aardweg:** Methodology, Investigation, Supervision, Writing – review & editing. **Anco Boonstra:** Conceptualization, Methodology, Investigation, Supervision, Writing – review & editing. **Elisabeth M.W. Eekhoff:** Conceptualization, Methodology, Investigation, Supervision, Writing – review & editing.

## Declaration of competing interest

The authors declare that they have no conflict of interest.
