# Supplementary material for: Deterioration of pulmonary function: An early complication in Fibrodysplasia Ossificans Progressiva
Source: Bone Rep. 2021 Feb 25;14:100758. doi: 10.1016/j.bonr.2021.100758 (PMC7972965; doi:10.1016/j.bonr.2021.100758)
Supplement: Supplementary Table 1 — Volume of heterotopic ossification in relation to the pulmonary function. [file mmc1.docx]

Supplemental data

Table 1.

|  | FEV1 (L, (% )) | FVC (L, (% )) | TLC  (L, (% )) | HO chest (cm^3^) | HO chest + back (cm^3^) | HO body (cm^3^) |
| --- | --- | --- | --- | --- | --- | --- |
| 001 | 2.05 (45) | 2.53 (47) | 3.90 (53) | 76 | 427 | 465 |
| 002 | 0.61 (23) | 0.61 (21) | n/a | 154 | 154 | 412 |
| 003 | 2.25 (64) | 2.38 (57) | 4.96 (93) | 68 | 104 | 146 |
| 006 | 0.94 (27) | 1.23 (31) | 2.92 (56) | 144 | 398 | 1131 |
| 007 | 1.47 (39) | 1.35 (35) | 2.31 (39) | 324 | 349 | 823 |

Abbreviations: FEV1 = forced expiratory volume in one second; FVC = forced vital capacity; TLC = total lung capacity, HO = heterotopic ossification; L = liters, % = percentage of predicted based on age, gender and ethnicity
